# Experiments with Barium Chloride1Read before the Montreal Veterinary Medical Association.

**Published:** 1896-03

**Authors:** J. J. McCarrey

**Affiliations:** M’gill University


### EXPERIMENTS WITH BARIUM CHLORIDE.[1]

By J. J. McCARREY,
M'GILL UNIVERSITY.

The following case-reports embody the results of some experiments recently made by the '96 Journal Club with barium chloride as a therapeutic agent in the treatment of colic. This drug was brought to the notice of the profession by Dickerhoff, who highly recommends the agent on account of its prompt and efficient action in producing peristalsis, and its low cost as compared with pilocarpine and eserine.

Case I. Bay gelding, aged, weight about twelve hundred pounds, an experimental subject. The animal in question received an intravenous injection of 1 gramme of barium chloride in solution. In four minutes the peristaltic murmur was much increased, followed by the evacuation of feces. In nine minutes another evacuation occurred, and again in twenty minutes. The pulse and temperature were unchanged throughout.

Case II. Bay mare, weight one thousand pounds, suffering from spasmodic colic. Was given an intravenous injection of 1 gramme of barium chloride in 8 c.c. of water. In three minutes there was considerable straining and increased uneasiness. In five minutes the animal defecated, the feces being hard in consistence. In thirty minutes after injection feces were again

[1] Read before the Montreal Veterinary Medical Association.

expelled, and at intervals of a few minutes for an hour, each evacuation being softer than the preceding. Straining was marked between evacuations, indicating violent peristalsis. In this case there was a slight increase in pulsations, respiration, and temperature. In three hours the animal showed no symptoms of colic, and the effect of the drug had apparently passed off.

Case III. Bay mare, aged. Received a subcutaneous injection of 1.2 grammes of barium chloride in 10 c.c. of water. One minute after injection the animal appeared uneasy. In fifteen minutes after she commenced to strain, but no evacuation occurred till half an hour after receiving the injection. After this at intervals of a few minutes evacuations occurred, continuing for about an hour and a half. No change in temperature or respirations, but the pulse was slightly accelerated.

Case IV. Brown gelding, aged, suffering from spasmodic colic. Received 1 gramme of barium chloride dissolved in about 10 c.c. of water intravenously. Feces and flatus passed in nine minutes, although straining was noticed about one minute after injection. Evacuations took place at intervals of a few minutes, continuing for half an hour, although there was an abatement of colicky symptoms immediately after the first evacuation.

Case V. Bay gelding, five years old, showing similar symptoms to preceding case. Received 1 gramme barium chloride dissolved in 10 c.c. of water intravenously in the jugular vein. Feces were not passed till twenty minutes had elapsed, but after that at intervals of five to fifteen minutes. This case did not recover from colic for three hours, although the violence of the symptoms were much lessened after the first evacuation.

Case VI. The subject in this case was a thoroughbred mare, five years old, and in about the fifth month of pregnancy. The animal was taken with colic at 9 A.M. Anodynes were given with warm-water injections. This treatment was continued throughout the day without any improvement. At 8 P.M., as a *dernier ressort*, it was decided to use barium chloride, the use of which had been delayed on account of the state of pregnancy in which the animal was in. At 8.15 P.M. an intravenous injection of 15 grains in 5 c.c. of water was given. At 8.19 the first symptoms of the action of the drug were noticed. The animal laid down and strained violently. Five minutes after flatus and a small quantity of almost liquid feces were passed.

Straining continued at intervals for half an hour without the passage of feces, and the colicky symptoms apparently aggravated. At 9 P.M. rectal injections of warm water were given, and an exploration revealed the fact that the colon was filled with feces, which were passed a few minutes later. The animal made a complete recovery.

